# Bacterial Molecular Signals in the *Sinorhizobium fredii*-Soybean Symbiosis

**DOI:** 10.3390/ijms17050755

**Published:** 2016-05-18

**Authors:** Francisco J. López-Baena, José E. Ruiz-Sainz, Miguel A. Rodríguez-Carvajal, José M. Vinardell

**Affiliations:** 1 Departamento de Microbiología, Facultad de Biología, Universidad de Sevilla, Avenida de Reina Mercedes, 6, 41012 Sevilla, Spain; rsainz@us.es (J.E.R.-S.); jvinar@us.es (J.M.V.); 2 Departamento de Química Orgánica, Facultad de Química, Universidad de Sevilla, Profesor García González, 1, 41012 Sevilla, Spain; rcarvaj@us.es

**Keywords:** soybean, *Sinorhizobium fredii*, *Bradyrhizobium*, Nod factors, type 3 secretion system, effector, exopolysaccharide, lipopolysaccharide, cyclic glucans, K-antigen polysaccharide

## Abstract

*Sinorhizobium* (*Ensifer*) *fredii* (*S. fredii*) is a rhizobial species exhibiting a remarkably broad nodulation host-range. Thus, *S. fredii* is able to effectively nodulate dozens of different legumes, including plants forming determinate nodules, such as the important crops soybean and cowpea, and plants forming indeterminate nodules, such as *Glycyrrhiza uralensis* and pigeon-pea. This capacity of adaptation to different symbioses makes the study of the molecular signals produced by *S. fredii* strains of increasing interest since it allows the analysis of their symbiotic role in different types of nodule. In this review, we analyze in depth different *S. fredii* molecules that act as signals in symbiosis, including nodulation factors, different surface polysaccharides (exopolysaccharides, lipopolysaccharides, cyclic glucans, and K-antigen capsular polysaccharides), and effectors delivered to the interior of the host cells through a symbiotic type 3 secretion system.

## 1. Introduction

Rhizobia are soil bacteria able to establish a symbiotic association with legumes in which a complex interchange of molecular signals is necessary for a successful infection. This molecular dialogue culminates in the formation of specialized plant structures, called nodules, on the roots and stems of the host plant. Within these structures, rhizobia differentiate into bacteroids able to fix atmospheric nitrogen into ammonia, which is used by the plant. In exchange, the legume provides an appropriate environment for bacterial growth.

Legume roots exude flavonoids responsible for the induction of genes dedicated to the synthesis and secretion of specific signal molecules called Nod factors (NF), which are responsible for nodule initiation and development. In addition to NF, there are other molecules such as surface polysaccharides and proteins secreted by bacterial secretion systems that also seem to play a very important role in the establishment of an efficient symbiosis with the host plant [1].

Soybean-nodulating rhizobia are alfaproteobacteria belonging to the genera *Bradyrhizobium*, *Mesorhizobium*, *Rhizobium* and *Sinorhizobium* (*Ensifer*) [2,3]. Taxonomic studies have shown that *Sinorhizobium* (*Ensifer*) *fredii* (*S. fredii*) is closely related to the alfalfa microsymbiont *Sinorhizobium meliloti*, although their host-ranges are remarkably different.

Soybean is a crop plant of enormous economic and agronomic interest [4]. *S. fredii* strains are potentially valuable for their use as soybean inoculants since they are able to grow much faster than bradyrhizobia. In addition to marked soybean cultivar specificity (discussed later), *S. fredii* strains show a remarkable broad host-range, being able to nodulate at least 79 different genera of legumes [5].

## 2. Nod Factors and Nodulation Genes

One of the first steps in the molecular dialog established between rhizobia and their legume host is the secretion of phenolic compounds, mainly flavonoids and isoflavonoids, by legume roots [6]. The most abundant flavonoids produced by soybean roots are the isoflavones daidzein, genistein, and coumestrol [7,8]. These plant signals are able to diffuse across bacterial membranes and are recognized by the bacterial protein NodD, a positive transcriptional regulator that, once activated by flavonoids, induces the expression of bacterial nodulation genes (*nod* genes) by binding to conserved bacterial promoters called *nod* boxes [9]. Bacterial *nod* genes code for enzymes involved in the synthesis and secretion of lipochitooligosaccharidic signal molecules called LCOs or Nod factors (NF), which in turn are recognized by plant LysM receptor-like kinases, inducing different responses required for the nodulation process such as root hair curling and nodule meristem initiation [10].

**Figure 1:**
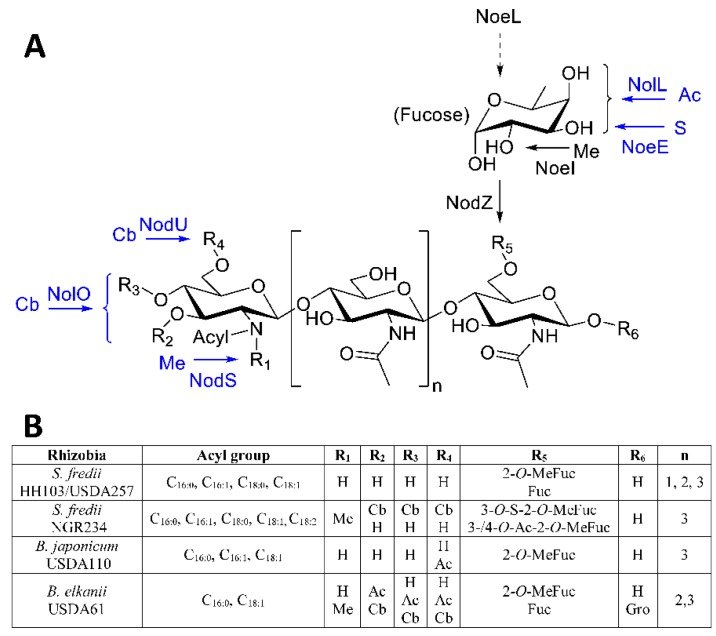
(**A**) Structure of nodulation factors produced by *Sinorhizobium fredii* HH103, USDA257, and NGR234 and Nod proteins involved in their decoration. Proteins marked in blue are only found in strain NGR234. Abbreviations of substituents are as follows: Ac, acetyl; Cb, carbamoyl; Me, methyl; S, sulfate; and (**B**) Comparison of Nod factors produced by different *S. fredii* strains, *Bradyrhizobium japonicum* USDA110, and *Bradyrhizobium elkanii* USDA61.

NF usually contain four or five β-(1→4)-linked *N*-acetyl-glucosamine (GlcNAc) residues and can harbor different decorations such as acetyl, sulfate, carbamoyl, arabinose, fucose, methylfucose. Each rhizobial strain produces not only one but a set of different NF, which are important for host-range determination [1,11]. Among the *S. fredii* strains, there is complete information about the LCOs produced by the *S. fredii* strains NGR234, USDA257 and HH103 (hereafter NGR234, USDA257 and HH103) [5,12,13,14]. The sets of NF produced by HH103 and USDA257, which induce the formation of nitrogen-fixing nodules in soybeans, are less diverse than that of NGR234, which is Nod^−^, with this legume (Figure 1). Thus, HH103 and USDA257 LCOs are oligomers of three to five β-(1→4)-linked *N*-acetyl-glucosamine (GlcNAc) residues bearing an amide bound fatty acyl residue (C16 or C18), saturated or unsaturated, on the non-reducing terminal GlcNAc residue, and a fucose or methylfucose modification at the C6 position of the reducing GlcNAc residue. In contrast, NGR234 NF are decorated not only with (methyl-)fucose but also with acetyl, carbamoyl, *N*-methyl, and sulfate groups. This high diversity of Nod factors is most probably one of the factors that accounts for the extraordinarily broad host range exhibited by NGR234 [5].

HH103, as well as USDA257, harbors in its symbiotic plasmid two different clusters involved in NF production: (i) the operon *nodABCIJnolO’noeI*, which is involved in the synthesis of the oligochitin skeleton and transport of mature NF; and (ii) the *nolK-noeL-nodZ-noeK* and *noeJ* cluster, required for the fucosylation of NF, and *noeI*, responsible for the methylation of the fucosyl residue [15,16]. NGR234 possesses additional *nod* genes involved in the acetylation (*nolL*), sulfation (*noeE*), carbamoylation (*nolO* and *nodU*), and *N*-methylation (*nodS*) of its NF [17,18,19,20] and HH103 lacks the *nolL* and *noeE* genes and contains truncated versions of the *nolO*, *nodU* and *nodS* genes [21]. HH103 and USDA257 NF are relatively simple and, in contrast to those of NGR234, lack acetyl and carbamoyl residues. Similar structures have been reported for other four different *S. fredii* strains isolated from different parts of China [22]. Thus, NF of strains B33, HWG35, WW10, and HH1 only carry fucosyl or *O*-methyl-fucosyl substitutions. On the other hand, strain HH17 produces a higher diversity of LCOs and harbors, in addition to (methyl-)fucosyl substitutions, acetylations and/or *N*-methylations [22].

The symbiotic plasmid (pSym) of HH103 harbors, together with the structural *nod* genes, several regulatory genes controlling nodulation such as two copies of *nodD*, *nodD1* and *nodD2* [23,24], *syrM*, and *ttsI* [25]. The symbiotic regulator *nolR* [26] is located in the chromosome.

Not all rhizobial genes controlled by *nod* boxes (NB) are involved in NF production. In HH103, only four out of the 15 NB identified in its genome are actually related with NF synthesis and secretion [21]: NB2 (*nolK-noeL-nodZ*), NB8 (*nodABCIJnolO’noeI*), NB12 (located upstream of two truncated genes, *nodS* and *nodU*), and NB19 (which drives the expression of the regulatory gene *syrM*). In addition, NB17 is responsible for the induction of *ttsI*, which codes for the transcriptional regulator of the symbiotic T3SS [25]. These data clearly indicate that legume-secreted flavonoids have a high impact in bacterial gene expression that exceeds the mere induction of NF production. A similar situation was reported for another soybean symbiont, *Bradyrhizobium japonicum* USDA110, whose genistein stimulon is composed by about 100 genes [27]. Further research is required to elucidate the putative symbiotic relevance of those genes that, being neither involved in NF production nor in type 3 secretion, are also regulated by NodD and plant-exuded flavonoids.

Detailed information about the NF produced by slow-growing soybean symbionts is also available [28,29] (Figure 1). Curiously, *B. japonicum* USDA110 produces LCO that are relatively similar to those of soybean-nodulating *S. fredii* strains, with the exception than they can also carry acetylations in the non-reducing end. In contrast, NF of *Bradyrhizobium elkanii* USDA61, as those of NGR234, can be decorated with methyl, acetyl, and/or carbamoyl substitutions in the non-reducing terminal GlcNAc residue [28]. On the other hand, the absence of *O*-methyl-fucose decorations in the HH103 NF by inactivation of the *noeL* gene does not have a drastic effect on the symbiotic interaction with soybean although it reduces the competitiveness to nodulate this legume [15]. In this sense, naked NF are enough to guarantee efficient nodulation with soybean, although the presence of (methyl)-fucose improves this interaction. The presence of additional decorations, such as carbamoylations, does not seem to disturb the symbiotic interaction with this legume.

Are NF absolutely required for soybean nodulation? In 2007, Giraud and coworkers [30] reported that some photosynthetic bradyrhizobia lacking the *nodABC* genes required for NF synthesis were able to nodulate some *Aeschynomene* species, demonstrating for the first time that a NF-independent symbiotic interaction was possible between a legume and a rhizobial strain. Very recently, Okazaki *et al.* [31] have shown that the T3SS allows a NF-deficient mutant of *B. elkanii* USDA61 to nodulate *Glycine max* cv. Enrei. An HH103 *nodD1* mutant, unable to produce NF, is able to slightly nodulate soybean when it overexpresses *ttsI* [32]. These results suggest that there is an alternative way to nodulate soybean based on the secretion of effectors through the T3SS.

## 3. Surface Polysaccharides

**Figure 2:**
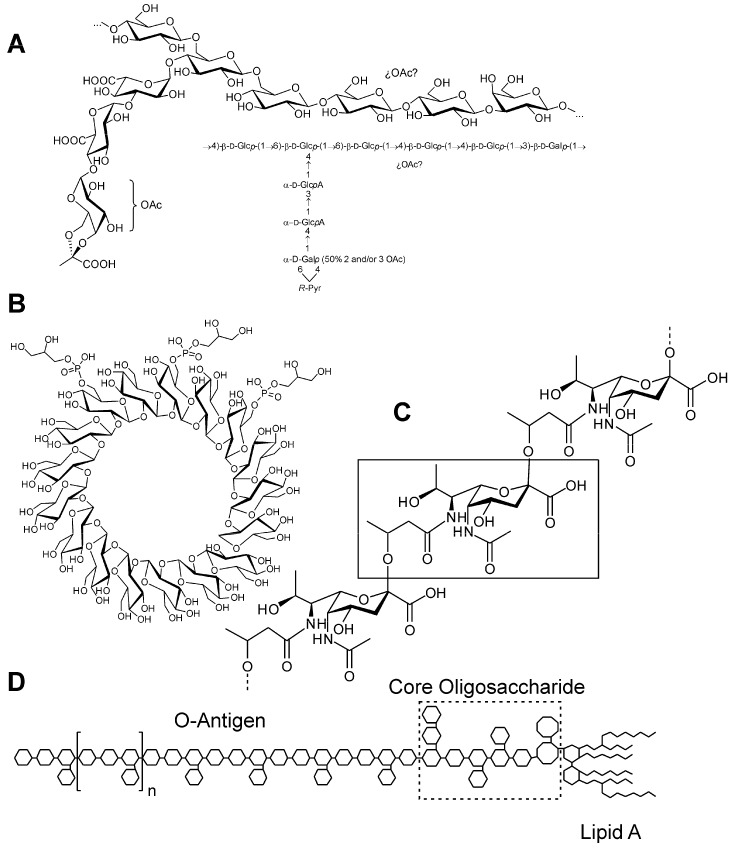
Structure of different *Sinorhizobium fredii* HH103 surface polysaccharides whose symbiotic relevance has been studied: (**A**) exopolysaccharide (EPS); (**B**) cyclic glucans (CG); (**C**) K-antigen capsular polysaccharide (KPS); and (**D**) lipopolysaccharide (LPS). ¿OAc?: *O*-acetyl group of unknown location. Lipid A: LPS moiety anchored to the bacterial external membrane.

In addition to Nod factors, different rhizobial surface polysaccharides are usually required for successful nodulation (Figure 2). Cyclic glucans (CG), exopolysaccharides (EPS), lipopolysaccharides (LPS), and capsular polysaccharides (KPS or K-antigens) are the main rhizobial polysaccharides investigated for their role in symbiosis [1,33]. The cell wall of Gram-negative bacteria is composed of the inner membrane (the cytoplasmic membrane) and the outer membrane, which are separated by the periplasmic space in which CG are located. EPS are located out of the cell, with little or no association with the outer membrane. Finally, LPS and, in *Sinorhizobium* strains, KPS are constituents of the outer membrane. These polysaccharides appear to play a crucial role, acting as signals required for the progression of the interaction and/or preventing host defense mechanisms [34].

## 4. K-Antigen Polysaccharides

K-antigen polysaccharides (KPS) are rhizobial acidic capsular polysaccharides analogous to group II of K-antigens described in *Escherichia coli* [35,36]. Among rhizobia, these polysaccharides were first described in *S. meliloti* and *S. fredii* [37,38], in which they are ubiquitous. Later, the presence of KPS has also been described in other rhizobia, such as *Mesorhizobium loti* and *Rhizobium tropici* [39,40]. In contrast to exopolysaccharides (EPS), whose structure varies at the species or biovar level, the KPS structure is strain-specific (Table 1). However, the presence of a dimeric repeating unit composed of one hexose and a 3-deoxy-d-*manno*-oct-2-ulosonic acid (Kdo) or a Kdo-derivative (Kdx) is commonly found in those *S. meliloti* strains that produce symbiotically active KPS, such as Rm41 [33,37,41]. By contrast, strain Sm1021 only contains a poly-Kdo that does not play any role in symbiosis [42]. In fact, poly-Kdo is also present, in addition to a symbiotically active form of KPS, in Rm41 as well as in several *S. fredii* strains [43], including HH103. The presence of a sugar-Kdx repeating unit has also been found in *S. fredii* strains that form nitrogen-fixing nodules with Asiatic soybeans (unbred soybean cultivars) but fail to effectively nodulate agronomically improved American soybean cultivars [37,41], as well as in NGR234 [44], which is Nod^−^, with soybeans. Curiously, other *S. fredii* strains, including HH103, which are Fix^+^ with both Asiatic and American soybean cultivars, produce KPS with structural motifs that do not follow the sugar-Kdx consensus [38,45,46,47]. Among these strains, the chemical structure of the HH103 KPS appears to be unique since it is a homopolysaccharide in which the repeating unit is a derivative of the pseudoaminic acid [45].

The fact that, up to date, the presence of hexose-Kdx in the KPS correlates with the Fix^−^ symbiotic phenotype with American soybeans, clearly suggest that KPS could participate in the determination of the symbiotic compatibility between *S. fredii* strains and agronomically improved American soybeans. However, as discussed below in this review, it is accepted that this compatibility is partially determined by the effector proteins secreted by a type 3 secretion system (T3SS) [11]. In fact, USDA257 mutants affected in the *nolWXBTUV* locus that encodes components of this secretion system gain the ability to nodulate American soybeans [49]. Interestingly, these mutants are reported to present clear changes in both LPS and KPS [41], although, unfortunately, to our knowledge, the exact nature of those changes has never been published.

The *S. meliloti* Rm41 strain-specific KPS, called K_R_5 antigen, is composed by glucuronic acid and a derivative of the pseudoaminic acid [37,41]. This strain contains three different genetic regions related to K_R_5 antigen production: *rkp-1*, *rkp-2* and *rkp-3* [50]. The *rkp-1* region is involved in KPS export and its encoded products participate in the synthesis and modification of a specific lipid carrier involved in secretion of this polysaccharide [51]; at least in *S. meliloti* 1021 this region is also required for secretion of the poly-Kdo present in *Sinorhizobium* strains [52]. The *rkp-2* region is composed by two genes, *lpsL* and *rkpK*, although only the latter, responsible for the synthesis of glucuronic acid, participates in the synthesis of the K_R_5 antigen [53]. Finally, the *rkp-3* region contains genes involved in KPS export and genes coding for enzymes responsible for the synthesis of the pseudoaminic acid derivative present in K_R_5 [49,54]. These three genetic regions are also present in HH103 and NGR234 [44,55,56,57,58], although only *rkp-1* and *rkp-3* have been proven to be related to KPS biosynthesis.

In *S. meliloti*, KPS and EPS are symbiotically equivalent for a successful nodulation with *Medicago sativa* (alfalfa) [59]. The situation in HH103 is clearly different. Thus, KPS-deficient *rkp-1* mutants are clearly impaired, although a few Fix^+^ nodules are still formed, in nodulation with soybean (determinate nodules) and pigeon pea (*Cajanus cajan*) and *Glycyrrhza uralensis* (indeterminate nodules) regardless of the EPS production. Therefore, in HH103 KPS and EPS are not symbiotically equivalent. Furthermore, a double KPS EPS HH103 mutant is still able to nodulate with legumes forming indeterminate nodules [55,58]. In NGR234 the absence of KPS causes a decrease in the bacterial symbiotic capacity with all the host plants tested: cowpea (*Vigna unguiculata*), *Flemingia congesta*, *Leucaena leucocephala* and *Tephrosia vogelii* [44]. Curiously, symbiosis with cowpea, which forms determinate nodules, is not affected by mutations in the *rkp-1* locus in HH103 [56], whereas NGR234 *rkp* mutants are severely impaired in nodulation with this legume.

The fact that HH103 nodulates both Asiatic and American soybeans allows the study of the importance of the KPS in the symbiotic interaction with these two kinds of soybean cultivars. In both plants, HH103 *rkp-1* mutants are able to induce the formation of a few Fix^+^ nodules showing normal morphology, invasion of nodule cells and proper nitrogenase activity [58], which indicates that a few root infections are able to overcome the impairment caused by the absence of KPS during the nodulation process. However, in American soybeans, the impairment appears to be more severe than in Asiatic cultivars, since in the former there is a stronger reduction in the number of Fix^+^ nodules and, in addition, many pseudonodules are formed. These data are the first clear indication that the HH103 KPS could play a role in the determination of cultivar-specificity.

On the other hand, HH103 *rkp-3* mutants, which are KPS deficient but also show alterations in the lipopolysaccharide (LPS), induce only pseudonodules on soybean, cowpea and pigeon pea, although most probably this symbiotic incapacity is mainly due to the LPS alterations rather than to the incapacity to produce KPS. Interestingly, HH103 *rkp-3* mutants are still able to induce the formation of Fix^+^ nodules in *G. uralensis* [57].

## 5. Exopolysaccharides

Exopolysaccharides (EPS) are homo- or heteromeric acidic polysaccharides secreted to the cellular environment and located on the cell surface. In contrast to capsular polysaccharides, EPS show little or no cell association [60]. EPS are widely distributed among bacteria and provide protection against different stresses such as desiccation or the presence of antimicrobial compounds, participate in attachment to surfaces and in biofilm formation and can also contribute to nutrient gathering [33,61]. The composition of EPS is highly variable. In rhizobia [60], EPS mainly contains common monosaccharides such as d-glucose, d-galactose, d-mannose, as well as d-glucuronic and d-galacturonic-acids. In addition, non-carbohydrate substituents such as *O*-acetyl groups or ketal-linked pyruvate and succinyl half ester groups are often detected. EPS can be produced as two forms of different molecular weights: High Molecular Weight (HMW, polymers of 10^6^–10^7^ Da) and Low Molecular Weight (LMW, monomers, dimers and trimers of the repeating unit) forms [61].

**Figure 3:**
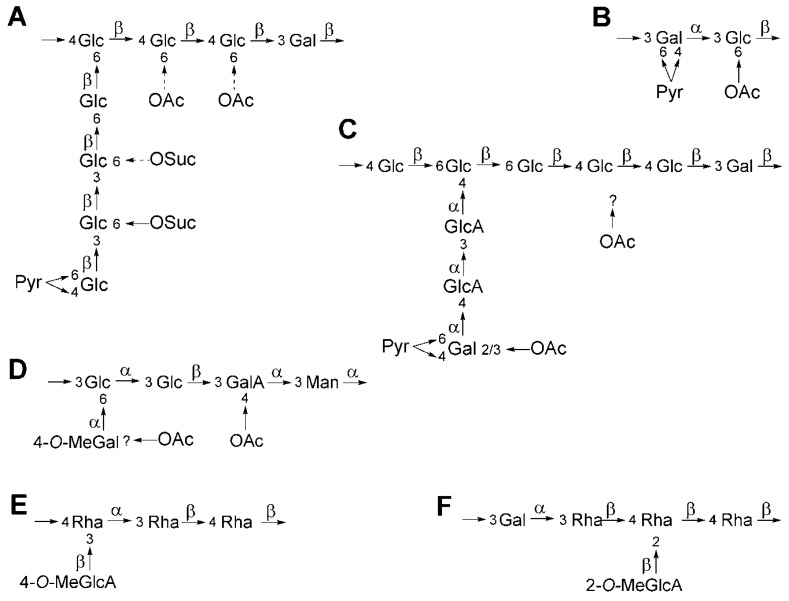
Structure of the EPS (and NPS) produced by different sino- and bradyrhizobia. *Sinorhizobium meliloti* 1021 EPS I (**A**) and II (**B**); *Sinorhizobium fredii* HH103 and NGR234 EPS (**C**); *Bradyrhizobium japonicum* USDA110 EPS (**D**); *Bradyrhizobium elkanii* USDA61 EPS/NPS (**E**); and *B. japonicum* USDA110 NPS (**F**). Dashed arrows indicate partial substitutions. α and β: α- and β-glycosidic linkages. ?: the position of the *O*-acetyl group is unknown.

The genetics of rhizobial EPS production has been extensively studied in *S. meliloti* and *Rhizobium leguminosarum* bv. *trifolii* [60,61]. *S. meliloti* produces two different kinds of EPS [62] (Figure 3), EPS I (succinoglycan), which is more related to the EPS produced by *S. fredii* strains [63], and EPS II (galactoglucan), produced under P starvation conditions. *S. meliloti* genes involved in EPS I production (*exo*/*exs*) are clustered in a ~35 kb region located on the largest megaplasmid, pSymB. The recent sequencing of the HH103 and NGR234 genomes [21,64] has revealed that these strains contain in their largest megaplasmid an identical *exo*/exs cluster, which is also very similar [63] to that of *S. meliloti* 1021. In contrast to *S. meliloti*, *S. fredii* strains do not produce a second EPS. Coherently, orthologs of the *S. meliloti* 1021 *wga*, *wgc*, *wgd*, *wge* and *wgg* genes, which are involved in the production of EPS II, are not present in the HH103 genome [21].

Recently, different roles of the HH103 EPS in free-living bacteria [63] have been reported. Thus, the absence of EPS in HH103 increases its osmosensitivity to NaCl and sucrose and decreases surface motility. This polysaccharide is the main responsible for the capacity of HH103 to attach to plastic and glass surfaces, but is not related to soybean roots attachment. In fact, *S. fredii* strains, in clear contrast to *B. japonicum*, are not able to bind soybean lectin, which indicates that the attachment of *S. fredii* and *B. japonicum* strains to soybean roots is mediated by different mechanisms. The different EPS structures of *S. fredii* and *B. japonicum* [65,66] (Figure 3) could account, at least partially, for the different capacity of these rhizobial species to attach to soybean roots and to recognize soybean lectin.

In addition to the EPS present in free-living cells, *Bradyrhizobium* produces a polysaccharide called NPS (nodular polysaccharide) in soybean root nodules. Most likely, NPS accumulates within the peribacteroid space. In *B. japonicum*, the composition of NPS (rhamnose, galactose and 2-*O*-methylglucuronic acid) and EPS (glucose, mannose, galactose, 4-*O*-methylgalactose and galacturonic acid) is different (Figure 3), in contrast to *B. elkanii*, which produces identical EPS and NPS composed of rhamnose and 4-*O*-methylglucuronic acid (Figure 3). Interestingly, *B. japonicum* and *B. elkanii* produce very different EPS but similar NPS, suggesting an important symbiotic role of NPS [33,67].

In rhizobia the EPS is also important for the establishment of effective symbiotic interactions with host legumes. In *Azorhizobium caulinodans*, the EPS forms a protective layer that prevents penetration of toxic H_2_O_2_ generated by its host legume, *Sesbania rostrata*, during invasion [34]. It has been traditionally accepted that the importance of bacterial EPS is much higher in symbiosis forming indeterminate nodules such as *S. meliloti*-*Medicago* or *R. leguminosarum* bv. *trifolii*-*Trifolium*, in which EPS-deficient mutants are defective in nodule invasion and nodules formed (Fix^−^) are consequently devoid of bacteria [68,69]. However, this is not always the case, since an HH103 *exoA* mutant is still able to induce nitrogen-fixing nodules on two plants forming indeterminate nodules: pigeon pea and *G. uralensis* [55,58]. On the other hand, in legumes forming determinate nodules, rhizobial EPS-deficient mutants are usually not or little impaired in symbiosis. Thus, an HH103 *exoA* mutant is fully effective with soybean and cowpea [55,56] and the same phenotype is observed for a *M. loti* R7A *exoB* mutant with *Lotus japonicus* [70]. In fact, in the case of HH103, the absence of EPS increases its competitiveness to nodulate soybean, suggesting that in this interaction the EPS is dispensable but somehow diminishes the capacity of HH103 to infect this plant. However, recent studies indicate that not the absence but alterations in the structure of the EPS of *B. japonicum* USDA110 or *M. loti* R7A lead to severe impairment in their symbiosis with soybean and *Lotus* respectively [70,71]. In fact, very recently a receptor-like kinase (EPR3) that directly binds EPS has been identified in *L. japonicus* [72]. This study shows that this receptor is able to distinguish between compatible and incompatible EPS and hence controls the passage of bacteria through the plant’s epidermal cell layer. This work provides strong evidence of the role of EPS as a molecular signal participating in root infection by rhizobia. On the other hand, the symbiotically relevant form of *S. meliloti*, NGR234 and *R. leguminosarum* bv. *trifolii* EPS is the LMW [73,74,75], suggesting again a role of EPS as a molecular signal that has to be recognized rather than the mere role of acting as a protective layer of polysaccharide.

## 6. Cyclic Glucans

Cyclic glucans (CG) are cyclic homopolymers of glucose residues. HH103 produces cyclic β glucans composed of 18 to 24 glucose residues without or with 1-phosphoglycerol as the only substituent [76] (Figure 2). Similar GC structures were described in *S. meliloti*, *S. fredii* and *M. loti* [77,78,79]. *B. japonicum* USDA110, however, produces a CG composed of 13 glucosyl residues distributed in a ring of 12 residues and one branched, β-(1→6) linked, glucosyl residue [80]. CG are mainly present in the periplasmic space although they can also be secreted to the extracellular environment and act as important factors in the formation of nitrogen-fixing nodules on host plants [81].

CG were first described as important pathogenic determinants in *Agrobacterium tumefaciens*. In rhizobia, the presence of GC was first described in *S. meliloti* [76,82]. Thus, mutants unable to produce CG were defective in root infection and only formed pseudonodules devoid of bacteria (Inf^−^ phenotype). Two genes, *ndvB* and *ndvA* (“*ndv*” refers to the impairment in nodule development shown by these mutants), were responsible for CG production and secretion. While NdvB is involved in CG biosynthesis, NdvA participates in its secretion to the periplasmic space. To date, all *S. fredii* strains investigated possess the *ndvA* and *ndvB* genes [21,64,83].

The inactivation of the rhizobial CG synthase (*ndvB* or *cgs*) provokes an Inf^−^ symbiotic phenotype in all cases studied [84]. For instance, an HH103 *ndvB* mutant, unable to produce CG, only formed pseudonodules on soybean (determinate nodules) and on *G. uralensis* (indeterminate nodules). Cowpea roots inoculated with the *ndvB* mutant formed nodule primordia but macroscopic structures, such as pseudonodules, were not observed [76]. Similarly, a NGR234 *ndvB* mutant also failed to induce macroscopic responses in cowpea roots [85]. As a consequence, the nodulation process induced by the *S. fredii ndvB* mutants is aborted at earlier stages in cowpea than in soybean. The HH103 *ndvB* mutant is neither impaired in NF production nor in bacterial survival in the cowpea rhizosphere. Therefore, the early disrupted nodulation process could be due to the absence (or miss-function) of an early bacterial signal associated to the absence of CG (CG may play important signal functions) or to a combination of pleiotropic disturbances in the bacterial surface associated with the absence of CG. In this sense, while the LPS and KPS were identical to those produced by the wild-type strain, an over production of an EPS of higher molecular weight bearing a higher level of substitutions (assigned as acetate and pyruvate substituents) was detected [76]. Whether these EPS alterations reduce the *ndvB* mutant symbiotic fitness remains to be elucidated.

Rhizobial *ndvB* mutants are affected in a diversity of bacterial functions, such as hypoosmotic adaptation, motility, attachment and infection [76,82,85]. However, the HH103 *ndvB* mutant is neither significantly affected in its attachment capacity to soybean roots [63] nor in its survival capacity under severe hypoosmotic conditions [76], indicating that HH103 CG might not be relevant for osmotic homeostasis.The biosynthesis of CG appears to be osmotically regulated in many rhizobial strains. Thus, CG production increases at low osmolarity [77,82].

There is no correlation between rhizobial CG structures and nodulation-specificity. For instance, *B. japonicum* and *S. fredii* share nodulation capacity with soybeans but while *B. japonicum* produces β-(1→6)-β-(1→3)-glucans, *S. fredii* produces β-(1→2)-glucans [76,80]. However, a *B. japonicum ndvC* mutant that produces an altered CG [the presence of β-(1→6)-linked glucosyl residues is severely reduced] only induces pseudonodules with soybeans [86].

Different reports have shown that CG could play a signaling role in the nodulation process [86,87]. For instance, the addition of purified CG increases nodulation of alfalfa roots inoculated with *S. meliloti* 102F34. However, the exogenous application of CG at the time of inoculation with a *S. meliloti* 102F34 *ndvB* mutant was ineffective in correcting the symbiotic impairment [88]. Although *S. meliloti ndvB* mutants form pseudonodules on alfalfa roots, bacterial pseudorevertants able to effectively nodulate alfalfa in the absence of CG can be isolated, indicating that CG might not be strictly required for nodule formation [88].

## 7. Lipopolysaccharides (LPS)

Bacterial lipopolysaccharides (LPS) are very complex glycolipid molecules that are part of the outer membrane of Gram-negative bacteria. The best characterized rhizobial LPS structures are those from *Rhizobium etli* and *Rhizobium leguminosarum* bv. *viciae* [89]. This complex structure can be divided into three different regions (Figure 2), called O-chain polysaccharide (or O-antigen, due to its antigenic properties), core oligosaccharide, and lipid-A. The lipid-A is the inner region of the LPS molecule that anchors the entire LPS to the bacterial outer membrane by means of its long fatty acyl moieties. This glycolipid usually contains a glucosamine disaccharide backbone that carries phosphoryl substitutions and/or other substitutions [90]. The lipid-A region is linked to the core oligosaccharide through an oligosaccharide-lipid linkage in which Kdo (3-deoxy-d-*manno*-2-oct-ulosonic acid) is involved.

The core oligosaccharide can be composed of up to 15 residues. The structure of the *R. etli* CE3 LPS-core has been completely determined [89]. Two core oligosaccharides are present; one is a trisaccharide consisting in two galacturonic acid residues and Kdo, and the other is a tetrasaccharide composed of galactose, galacturonic acid, mannose and Kdo [41,89,90,91]. The core oligosaccharide of USDA257 LPS is composed of Kdo, glucose, galactose, glucuronic acid, and galacturonic acid [92]. This carbohydrate composition is similar to that described for the LPS core of *S. meliloti* 1021 [93].

Rhizobial O-chains are the most variable part of the LPS molecule and it is generally constituted by a polymer of repeating units consisting of one to five residues. The monosaccharide composition of the repeating unit is very variable as well as the types of substituents (decorations) present, rendering an enormous structural diversity. The R. etli CE3 O-chain repeating unit contains glucuronic acid, fucose, and 3-*O*-methyl-rhamnose [94]. The structures of other *O*-polysaccharides are well known for R. leguminosarum bv. trifolii, R. tropici CIAT899, and Mesorhizobium huakuii [91,95,96]. The structures of the *S. fredii* and *S. meliloti* O-chains appear to be different from those of *Rhizobium* and *Bradyrhizobium* [92]. Composition analyses of sinorhizobial O-antigens indicate that they might be homopolymers of low antigenicity, the core being the dominant antigenic region of the whole LPS molecule [97].

Although the structure of the HH103 LPS has not been determined, Kdo, glucose, glucuronic and galacturonic acids, and 5-acetamido-3,5,7,9,-tetradeoxy-7-(3-hydroxybutyramido)-l-*glycero*-l-*manno*-non-2-ulosonic acid have been detected (our own unpublished results). The latter is also a component of the HH103 KPS. This fact explains why *S. fredii* HH103 mutants affected in genes involved in the biosynthesis of the KPS repeating unit also show alterations of the HH103 LPS [57].

Since rhizobia can live in soils (free-living state) or inside legume nodules (endophytic state), they must adapt to different environmental changes. Such adaptation requires changes in the rhizobial surface polysaccharides, including LPS. The identity of the bacterial surface polysaccharide and the nature of the structural changes vary among the different rhizobia–legume symbiotic interactions. Although the symbiotic functions of rhizobial LPS are not totally understood, they can be mainly divided in a direct role in symbiosis, such as root infection, bacterial release into nodule cells, symbiosome multiplication, and bacteroid formation or promotion of symbiosis due to functions associated to protection of rhizobia against plant defense compounds or by inhibiting host defense mechanisms [89].

Rhizobial LPS mutants, such as those of *S. meliloti*, exhibit a pleiotropic phenotype that results from alterations in the bacterial outer membrane. These phenotypes can include rough colony morphology, increased sensitivity to detergents and alterations in phage sensitivity [93]. With respect to symbiosis, many rhizobial LPS mutants show diverse impairments, ranging from the only induction of pseudonodules devoid of bacteria [98] to the formation of early-senescent nitrogen-fixing nodules [99,100]. Mutations in genes controlling the synthesis of the LPS can also alter bacterial host-range of nodulation. For instance, *S. meliloti* 2011 *lpsB* mutants induce effective nodules on alfalfa but fail to establish a symbiosis with *M. truncatula* [101].

Although the HH103 *greA*, *lpsB* and *lpsE* mutants induce the formation of numerous pseudonodules, they are still able to induce the formation of some nodules of normal external morphology in soybean [100]. The infected cells of these nodules showed symptoms of early symbiosis termination and lytic clearance of bacteroids. These cells also showed very thick walls and accumulation of phenolic-like compounds, indicating the induction of plant defense reactions at late symbiotic stages. *B. japonicum* LPS mutants lacking the O-antigen polysaccharide were impaired in early nodulation stages, since they failed to infect soybean root cells and were unable to fix nitrogen [98]. Therefore, rhizobial LPS can be important in early and late symbiotic stages and they could play a role in preventing host cell defense reactions.

In summary, data available so far indicate that the symbiotic relevance of each rhizobial polysaccharide appears to be related to each specific bacterium–legume interaction rather than to the type of nodule, determinate or indeterminate, formed by the host plant.

## 8. The Type 3 Secretion System

The type 3 secretion system (T3SS) has been intensively studied in the last years due to its importance in the promotion of diseases in plants and animals. The secretion machinery extends from the inner to the outer membrane crossing the periplasmic space and is prolonged to the exterior through an extracellular cylindrical appendage or *pilus*, which forms a narrow conduit necessary for secretion and injection of proteins, called effectors, directly into the host cells. The term effector groups a set of proteins secreted through the T3SS that exert their function mainly into the host cell [102], where they alter host signaling and suppress plant defenses, providing a beneficial environment for bacterial multiplication [103]. However, the effectors can also be recognized by specific plant resistance (R) proteins and induce strong defense responses, frequently associated with a localized hypersensitive response (HR), to block infection [104].

In sinorhizobia, the first complete sequence of the genes involved in the biosynthesis of the T3SS, grouped in the *tts* region, was obtained after sequencing the symbiotic plasmid of NGR234 [105]. However, first evidences about the existence of rhizobial genes that could be related to the biosynthesis of the T3SS were obtained after the observation that USDA257 could secrete a number of proteins to the extracellular medium upon induction with flavonoids and in the presence of NodD1. These proteins, initially called SR (signal responsive),were later called Nops (nodulation outer proteins) [106,107,108]. As previously mentioned, the USDA257 T3SS is involved in the capacity of this strain to nodulate specific soybean cultivars. Thus, USDA257 induces the formation of nitrogen-fixing nodules in Asiatic soybeans but not in American cultivars. USDA257 mutants unable to secrete Nops gain the capacity to nodulate American soybeans [49] (Table 2). However, there are other *S. fredii* strains, such as HH103, that possess a functional T3SS and can naturally nodulate both soybean cultivars (Table 2) and also others, such as NGR234, which cannot nodulate this plant [5,25,109].

In the last years, sequencing of the complete genomes of the three most representative sinorhizobial strains, namely HH103, USDA257 and NGR234, has provided plenty of information about the genetic organization of the secretion system, as well as the identification of a second T3SS, regulation of the expression of the T3SS-related genes, and the presence of putative effectors [21,64,83].

To date, many sinorhizobial proteins secreted through the T3SS have been identified: NopA, NopB, NopC, NopD, NopJ, NopL, NopM, NopP, NopT, and NopX [118] (Table 3). It is necessary to differentiate between those proteins that are components of the extracellular appendages and hence are found in the extracellular medium in protein extractions and those that can be considered "putative" effectors until their direct secretion to the host cytoplasm is demonstrated. Thus, NopA and NopB are the main components of the extracellular appendage or T3SS-*pilus* of NGR234. NopX is also found in these structures but its function seems to be the translocation of proteins to the interior of the host cell [116,119,120,121].

On the other hand, NopC, NopD, NopJ, NopL, NopM, NopP, and NopT can be considered putative effectors. While NopD, NopJ, NopM, and NopT are similar to effectors found in different animal and plant pathogens, NopL, NopP, and NopC are specific for rhizobia [122].

The role of Nops in symbiosis is the consequence of the action of a certain cocktail of proteins that specifically function in a host plant. Thus, the absence of Nops can be highly beneficial, have no effect or be detrimental for the symbiotic interaction. While the negative effects are due to the action or the recognition of certain effector or effectors, the beneficial effects are the result of the effects of different. In the most extreme situations, the recognition of an effector completely blocks nodulation.

**Figure 4:**
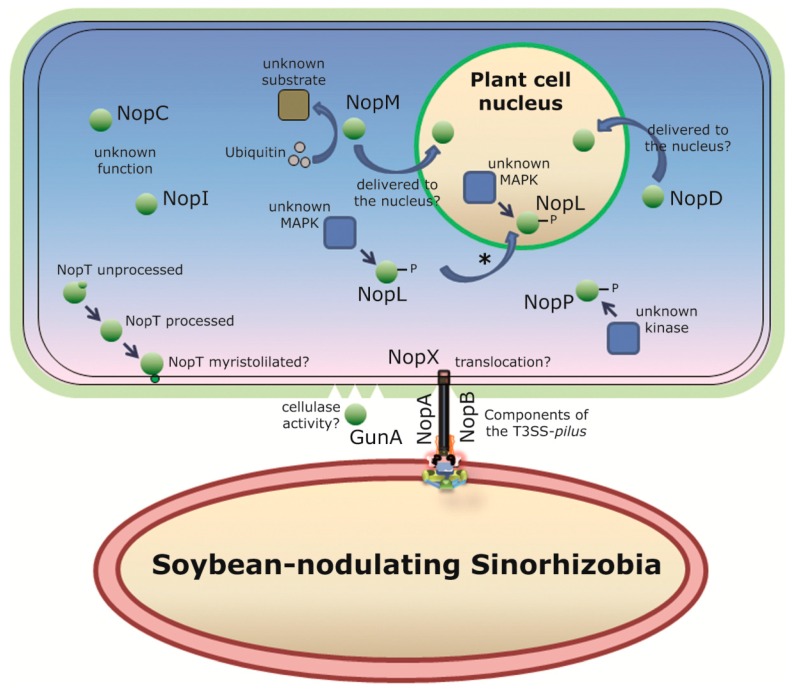
Functions and localization in the plant cell of different sinorhizobial nodulation outer proteins. Potential functions are discussed in the main text. Proteins secreted by the T3SS (Nop) are in green. The figure also shows NopA and NopB, main components of the extracellular appendage or *pilus*, and NopX, which probably forms a pore through which effectors enter the plant cytoplasm. * NopL could be phosphorylated by a cytoplasmic MAPK and then migrate to the nucleus or it could be delivered to the nucleus and be phosphorylated by a nuclear MAPK.

The nodulation outer protein NopM belongs to the IpaH-SspH-YopM family of effectors found in animal pathogens that travel to the nucleus of the host cell [123] (Figure 4). This protein was first described as secreted by the T3SS of HH103 [124]. This strain possesses two identical copies of *nopM*, termed *nopM1* and *nopM2* [21]. In rhizobia, NopM functions as a NEL domain E3 ubiquitin ligase and it seems to dampen the flg22-induced ROS burst in *Nicotiana benthamiana*, suggesting a role in MAMP-triggered immunity [125]. In symbiosis, the NGR234 *nopM* mutants showed reduced nodulation on *Lablab purpureus* but induced more nodules on *Pachyrizus tuberosus* [125,126]. The symbiotic effect of the inactivation of *nopM* in soybean-nodulating rhizobia has not been studied yet.

NopD is secreted through the HH103 T3SS and shows homologies to XopD from *Xanthomonas campestris*, to the hypothetic proteins Blr1693 and Bll8244 from *B. japonicum* and to Msi059 from *M. loti* R7A, a protein secreted through the type 4 secretion system [124]. Secretion of the *B. japonicum* Bll8244 through the HH103 T3SS has also been confirmed, indicating that effectors from different rhizobial strains are interchangeable [127]. The C-terminal region of these proteins shows homology to the catalytic domain of members of the family of C48 cysteine proteases involved in the de-ubiquitinisation of eukaryotic proteins. XopD possesses peptidase/isopeptidase activity *in planta* and is delivered to the interior of the plant nucleus (Figure 4). The targets for this effector are SUMO (Small Ubiquitin-like Modifiers) proteins involved in the regulation of the activity of diverse transcriptional factors that coordinate the expression of genes necessary for plant development and for plant responses to the environment, such as abiotic stress, adaptation to changes in the environment, plant responses mediated by ABA (abscisic acid) or defense responses against pathogens [128].

Recent works have shown that the inability of some *B. japonicum* strains, such as Is-1, to nodulate *Rj2* soybean cultivars (e.g., Hardee) is due to the presence of a protein with a C48 peptidase domain [129]. In addition, [130] showed that the inability of *B. elkanii* USDA61 to nodulate *Rj4* soybean cultivars is also partially mediated by a protein possessing this domain. In both cases, the protein candidates for the determination of nodulation-specificity in *Rj2* and *Rj4* soybeans are homologous to the HH103 NopD. However, to our knowledge, HH103 and also USDA110 are compatible with all *Rj* soybean cultivars. Therefore, more efforts are necessary to determine the exact mechanism underlying the recognition of these effectors by soybean R proteins.

NopJ and NopT are homologous to Avr proteins that are determinants of virulence in phytopathogens. NopJ belongs to the family of YopJ effectors that have a C55 domain with cysteine protease activity. This family includes many Avr proteins from phytopathogens able to induce the hypersensitive response (HR). The acetyl-transferase activity of these effectors blocks phosphorylation of MAP-kinases by acetylation of the sites of phosphorylation. The absence of the NGR234 NopJ has no effect on nodulation of *Tephrosia vogelii* and *P. tuberosus* but is beneficial for nodulation of *L. purpureus* and *Crotalaria juncea* [126].

NopT belongs to the family AvrPphB/YopT of proteins harboring C58 domains with cysteine protease activity [131]. As some members of this family, NopT has cysteine protease and autoprocessing activities [126,132,133] (Figure 4). Transient expression of NopT in nonhost *Nicotiana* plants triggered cell death, suggesting that the tobacco immune system is recognizing this protein as pathogenic [132,133]. Inactivation of *nopT* had a negative effect on nodulation of *T. vogelii* and common bean (*Phaseolus vulgaris*) and a positive effect on *C. juncea* [132]. Although NopT1 and NopT2 from *B. japonicum* USDA110 have been characterized [133], the effect of the mutation of *nopT* on soybean nodulation has not been studied yet. Interestingly, both HH103 and USDA257 possess a copy of the *nopT* gene (Table 3).

Both NopL and NopP are substrates for plant kinases [134,135] (Figure 4). Secretion of the USDA257 NopP to the interior of cowpea root cells has been confirmed indicating that this Nop can be considered a real effector [136]. With respect to soybean nodulation, the absence of the HH103 NopP induced the formation of more nodules on the American soybean cultivar Williams82 (Table 2). This improvement in nodulation efficiency is associated with a reduction in the expression of *GmPR1*, a common marker for the SAR response [117].

It has been proposed that NopL, once delivered to the interior of the host cell, would modulate signal transduction pathways regulated by MAP-kinases that culminate in the expression of PR proteins [137]. Some reports also indicate that NopL is directly involved in common bean nodule senescence [138]. Phosphorylation sites for NopL have been identified and recent works have shown that NopL is localized to the plant nucleus of onion cells (Figure 4) where it forms a complex with SIPK, a tobacco MAP-kinase. NopL is multiply phosphorylated by SIPK indicating that NopL can be hyperphosphorylated by MAP-kinases [139].

Together with NopL and NopP, NopC is also considered rhizobium-specific since no homologs have been detected in other bacteria. Translocation of NopC to the interior of soybean root cells has been reported, indicating that this protein can also be considered an effector. With respect to the role of NopC in symbiosis, its absence is detrimental for soybean and cowpea nodulation [113] (Table 2).

The analysis of the genome of HH103 revealed the presence of two novel “potential” nodulation outer proteins, NopI and GunA [21]. Further work is necessary to determine whether these proteins are secreted through the T3SS or if they are delivered to the interior of the host cell by means of the T3SS and also their role in symbiosis with soybeans and other host plants.

Increasing evidences indicate that rhizobia can be recognized by the plant immune system, blocking the infection in some cases or reducing nodulation efficiency [140]. In this context, the rhizobial T3SS could play an essential role in the suppression of these defense responses to promote nodulation. For instance, inoculation of soybean plants with HH103 mutants unable to secret Nops increases the expression of *GmPR1* in soybean roots, but also in leaves, suggesting that the defense response is not local but systemic. The higher expression of the soybean *GmPR1* correlates with an increase in salicylic acid (SA) production and with a decrease in nodule number. Therefore, Nops seem to suppress the plant defense responses triggered by identification of rhizobia in the early stages of symbiosis [32,117].

## 9. Conclusions and Perspectives

The molecular basis of symbiotic specificity in the rhizobium–legume interaction is far from being fully understood. *S. fredii* and *S. meliloti*, regardless their close phylogenetic relationship, differ in the width of their host-ranges, extremely broad and extremely narrow, respectively. At least part of these differences are due to the different range of flavonoids able to activate *nod* gene transcription in these sinorhizobial species. *S. fredii* NodD1 proteins only require the presence of a hydroxyl group at position C7 of the flavonoid skeleton to activate *nod* expression [11,24], whereas the different NodD proteins from *S. meliloti* have more exquisite requirements for an effective *nod* gene induction [141]. This fact appears to be the rule in other rhizobia–legume interactions such as those of the *Mesorhizobium* genus with different host legumes [142]. In addition, NF produced by broad host range rhizobia are simpler than those synthesized by narrow host range rhizobia and because of this the former can be effectively recognized by plant receptors of a high number of legumes.

Bacterial surface polysaccharides can also play important roles in the effectiveness and specificity of symbiosis. With the exception of CG, which seems to be essential in all the rhizobia–legume interactions tested so far, the effect of variations in LPS structure or even the absence of EPS or KPS appears to be specific for each symbiotic couple [29,55,56,57,58,59].

Some, but not all, rhizobia harbor a symbiotic T3SS to deliver effector proteins into host cells [122]. In the case of soybean-nodulating rhizobia, the T3SS plays a key role in cultivar specificity and symbiotic efficiency. More efforts are necessary to determine the exact role of each effector in symbiosis, to detect plant targets for each effector and to identify those plant processes regulated by the T3SS-secreted effectors. This information will be very useful to select the best soybean inoculants and should be taken into consideration in future soybean plant breeding projects.

Soybean is a legume with a marked cultivar specificity behavior. In the *S. fredii*-soybean interactions, the *Rfg1*/*Rj2* locus, present in American but not in Asiatic soybeans [143], impedes nodulation with a reduced group of sinorhizobial strains [144]. Although this incapacity is thought to be exclusively due to a still unknown effector secreted through the T3SS [145], we cannot discard that other factors, such as the KPS, could be playing a role in this issue.

The repertoire of rhizobial molecular signals involved in symbiosis can even be higher than expected. Genomic analyses of broad host range rhizobial strains, such as NGR234 and HH103 [21,64] show that their *nod* regulon comprises a large variety of genes, in addition to those devoted to NF production. Transcriptomic and proteomic analyses should increase our knowledge about the complete set of signals triggered by rhizobia when they enter in contact with the appropriate plant signals.

## Figures and Tables

**Table 1:** KPS structures of Sinorhizobia.

Species	Strain	KPS Structure [Reference]	Symbiotic Phenotype with Asiatic/American Soybeans
*Sinorhizobium meliloti*	Rm41	[-β-GlcA→Pse5N(3-OH-But)7NAc-]*_n_* [38]	NA
	Sm1021	[→7)-β-Kdo*p*-(2→]*_n_* [42]	NA
*Sinorhizobium fredii*	USDA201	[-α-Gal→β-Kdo→2-*O*-Me-α-Hex→β-Kdo-]*_n_* [38]	Fix^+^/Nod^−^
	USDA205	[→3)-α-d-Gal*p*-(1→5)-β-Kdo*p*-(2→]*_n_*	Fix^+^/Nod^−^
		[-2-*O*-MeMan*p*→β-Kdo-]*_n_* [37]	
	USDA208	[-α-Gal→β-Kdo-]*_n_* [38]	Fix^+^/Nod^−^
	USDA257	[→3)-β-d-Man*p*-(1→5)–β-Kdo*p*-(2→]*_n_*	Fix^+^/Nod^−^
		[→3)-β-d-2-*O*-MeMan*p*-(1→5)–β-d-Kdo*p*-(2→]*_n_* [48]	
	NGR234	[-β-Glc→Pse5NAc7NAc-]*_n_* [38]	Nod^−^/Nod^−^
	HH103	[→3′)-α-Pse5NAc7(3-OH-Bu)-(2→]*_n_* [45]	Fix^+^/Fix^+^
	HH303	[Rha, GalA]*_n_* [38]	Fix^+^/Fix^+^
	B33	[→6)-4-*O*-Me-α-d-Glc*p*-(1→4)-3-*O*-Me-β-d-Glc*p*A-(1→]*_n_* [46]	Fix^+^/Fix^+^
	HGW35	[→6)-2,4-di-*O*-Me-α-d-Gal*p*-(1→4)-β-d-Glc*p*A-(1→]*_n_* [47]	Fix^+^/Fix^+^

NA = Not applicable.

**Table 2:** Effect of the mutation of the *Sinorhizobium fredii* strains HH103 and USDA257 *tts* genes on soybean nodulation.

*tts* Gene Mutated	Function	Symbiotic Phenotype in American or Asiatic Soybean Cultivars	
		HH103	USDA257
*rhcJ*	T3SS machinery	American and Asiatic: reduced nodule number, mass of nodules and plant-top dry mass [109]	American: Fix^−^ to Fix^+^ [49]
*rhcC1*	T3SS machinery	–	American: Fix^−^ to Fix^+^ [49]
*nolU*	T3SS machinery	–	American: Fix^−^ to Fix^+^ [49]
*rhcL*	T3SS machinery	–	American: Fix^−^ to Fix^+^ [49]
*rhcN*	T3SS machinery	–	American: Fix^−^ to Fix^+^ [48,110]
			Asiatic: negative effect [110]
*y4yA*	Unknown	–	American and Asiatic: no effect [111]
*y4yB*	Unknown	–	American and Asiatic: no effect [112]
*y4xP*	Cysteine synthase	–	American and Asiatic: no effect [112]
*nopA*	T3SS *pilus*	American: reduced nodulation [113]	American: Fix^−^ to Fix^+^
			Asiatic: reduced nodulation [114]
*nopB*	T3SS *pilus*	–	American: Fix^−^ to Fix^+^ [49]
			Asiatic: negative effect [115]
*nopX*	T3SS *pilus*/effector translocation?	American: reduced nodulation [116]	American: Fix^−^ to Fix^+^ [49]
			Asiatic: delayed nodulation [116]
*nopC*	Effector	American and Asiatic: reduced nodulation [113]	–
*nopP*	Effector	American and Asiatic: increased nodule number and plant-top dry mass [117]	-

**Table 3:** Nodulation outer proteins identified in the *Sinorhizobium fredii* strains HH103, USDA257 and NGR234.

Nop	Size (kDa)	Function	Detected in Induced Culture Supernatant *			Gene Present in the Sequenced Genome		
			HH103	USDA257	NGR234	HH103	USDA257	NGR234
NopA	~6	T3SS *pilus*	Yes	Yes	Yes	Yes	Yes	Yes
NopC	~11	Effector	Yes	Yes	Yes	Yes	Yes	Yes
NopB	~21	T3SS *pilus*	Yes	Yes	Yes	Yes	Yes	Yes
NopI	~27	Putative effector	NC	NC	-	Yes	Yes	No
NopT	~28	Putative effector	NC	NC	Yes	Yes	Yes	Yes
NopJ	~29	Putative effector	-	-	ND	No	No	Yes
NopP	~32	Effector	Yes	Yes	Yes	Yes	Yes	Yes
NopL	~37	Putative effector	Yes	NC	Yes	Yes	Yes	Yes
NopX	~60	Translocation	Yes	Yes	Yes	Yes	Yes	Yes
NopM	~60	Putative effector	Yes	NC	Yes	Yes (x2)	Yes	Yes
NopD	~150	Putative effector	Yes	NC	–	Yes	Yes	No

* Detection using specific antibodies or mass spectrometry; NC: Not confirmed; ND: Not detected.
